# Obstetrics and Gynæcology

**Published:** 1880-05

**Authors:** 


### OBSTETRICS AND GYNÆCOLOGY.

THE DIAGNOSIS OF LARGE OVARIAN TUMORS.—In No. 1 *Centralblatt, für Gynäkologie* for the present year, Dr. B. S. Schultze, of Jena, records two illustrative cases in support of the value of the method of diagnosis recommended by him in No. 6 of the same journal in 1879, and already reported in our retrospect, vol. xxiv., p. 1131. This method consists in putting the patient deeply under chloroform, subjecting the tumor to alternate elevations and depressions and displacements from side to side by means of an assistant grasping it through the abdomen, whilst the examiner practices the abdomino-rectal manipulation, and is able to satisfy himself of the presence or absence of a uterine or ovarian connection with the tumor, the length of the pedicle, the character of it as to breadth and thickness, etc. Two extremely important cases are recorded, in which the diagnosis of an ovarian tumor, and the exact nature of its pedicle and source of origin, was readily made out by Schultze, notwithstanding that subsequent operation proved that the tumor had the most extensive adhesion to the abdominal wall, the omentum, colon, etc. Schultze argues that the application of this method as a means of exact diagnosis is wider than he had expected, and that the most extensive adhesions do not destroy its value. It therefore, according to him, will enable the operator to proceed in many otherwise doubtful cases directly to operate, when he would have, without it, required to make an exploratory examination.

ON THE ELUCIDATION OF THE INDICATIONS FOR TREATMENT OF ANTI- AND RETRO-VERSIONS AND OF ANTI- AND RETRO-FLEXIONS OF THE UTERUS, is the subject of No. 176 in Volkmann's *Collection of Clinical Lectures* by Schultze, and is abstracted in No. 2 of the above-mentioned *Centralblatt* for 1880. The author briefly explains his position on the question of versions and flexions. Generally, the importance of changes of position is asserted, but care is taken to guard against the theory that the same etiology and the same treatment is to be held as self-evident in the case of retroflexions and anteflexions and versions. The intra-uterine stems are altogether rejected. Whilst in retroflexions fixation of the fundus is secondary as an etiological influence, relaxation of Douglas's folds primary, in anteversion we find the reverse of the fact, viz., the pathological shortening of Douglas's folds to be the primary etiological influence. Some diagrammatic drawings indicate in the most simple and clear manner the views of the author. The legend of obstructive dysmenorrhœa, which has legalized so many fatal therapeutic procedures, is to be utterly rejected. That flexion of itself can condition stenosis the author emphatically denies. The hope is expressed that this view, stated authoritatively, will contribute to definitely liberate German gynæcology from the fetters of imported opinions unfortunately too readily accepted. In anteversions and anteflexions the author sees generally no indication for mechanical treatment. It is possibly a mistake to attempt to replace even retroflexions and retroversion with the pessary. We must first replace the uterus by hand; then the pessary has the single object and the single aim of retaining the now normally situated uterus in its normal position.

DISEASES OF BLOOD-VESSELS OF THE OVARY IN RELATION TO THE GENESIS OF OVARIAN CYSTS, is the subject of a highly interesting and original contribution to the January number of the *American Journal of Obstetrics* by Dr. Noeggerath of New York. The object of the paper is to demonstrate that the development of a certain percentage of ovarian cysts is due to certain alterations and enlargements of blood-vessels, and thus is formed a variety which the author thinks ought to be called *angioma cysticum*. The conclusion arrived at in the paper is founded upon careful microscopic examination of pathological ovaries, especially of six ovaries removed by Battey's operation from young girls. Dr. Noeggerath found that the structures usually described as *corpora albicantia* were frequently due to enlargement and degeneration of blood-vessels. The coats of the vessels are enlarged, with diminution, though rarely obliteration, of their lumina. The process commences apparently with œdema of the intima or adventitia, whilst the single fibers of the connective tissue element become individually indistinct, melting together, as it were, and ultimately appearing like an aggregation of very finely split up white elastic tissue fibrils glued together. With time the nuclei grow thinner and shorter, assuming shapes occasionally like what are met with in myxomatous tissue. The process could be traced, passing through different stages, from a period when the loops of blood-vesels could be distinctly made out to the time when nothing could be detected but a homogeneous mass. In the small arteries there could be detected a hyaline degeneration of the connective tissue of the intima and of the intermuscular connective tissue, and also a growth of the cellular elements of the media both in number and in size, with the effect of almost entirely obliterating their lumina. The structure formed by degeneration of the blood-vessels of a vascular territory, although exceedingly like a corpus luteum, is capable, Dr. Noeggerath says, however, of being differentiated from it by careful examination. The cells comprising the corpus luteum are deposited in meshes of irregular shape, and are more or less spherical; the degeneration of arteries, on the other hand, has its cells deposited in interstices resembling an oblong rhomboid. The cells comprising a corpus luteum are coarsely granular and yellowish; those of the arterial degeneration are only slightly granular. On further development the cellular element of the corpus luteum gradually gives way before the growth of the connective tissue part of it, the degeneration loses its fibers by atrophy, owing to the growth of single cells. In consequence we have first the formation of an irregular cavity in the center, containing broken up cells. Gradually all the cells comprised within the group of disorganized vessels become disaggregated, softened, and dissolved. Exosmosis adds to the now increased serous portion, and there results a small cyst whose walls consist to a large extent of normal ovarian tissue. These cysts are not lined with epithelium, and are probably of slow growth. In further developing the subject Dr. Noeggerath considers other changes which take place in the coats of ovarian vessels and finally result in the formation of cystic cavities. One mode of development he attributes to a kind of endarteritis destruens. Here at the commencement a large number of vessels, chiefly arteries, are found closely aggregated together. These have the peculiarity that the elements of their media have increased in number and in size, so as to make the intima appear in a state of atrophy. These ultimately develop into well-formed layers of muscular tissue. Still the several bundles are so arranged that we can without difficulty recognize their development from vessels. The characteristic peculiarity of these masses is that a large number of fissures of oblong shape, and each containing a cell, are arranged concentrically round them. These are larger the nearer the center or lumen of the vessel they are placed, and they tend to enlarge so as to break up the tissues and empty their contents into the lumen, which thus becomes enlarged and dilated into a cyst. Dr. Noeggerath is further driven to conclude that in a certain number of cases those epithelial tubes found in ovaries as precursors of ovarian cysts are not derived, as Waldeyer, De Sinety, Manassez, etc., believe, from germinal epithelium, but from the tissue comprising the capillary blood-vessels, as he finds the microscopical characters of undoubted vascular changes seen by himself come exceedingly near in general appearance to these so-called tubes as figured by Waldeyer.

A CASE OF ACUTE SPONTANEOUS INVERSION OF THE UTERUS OCCURRING AFTER ABORTION DURING THE FIFTH MONTH OF PREGNANCY, is recorded by Dr. Scott of Philadelphia, in the same journal for January. The details of the case are given with some minuteness, and are interesting as demonstrating that the inversion occurred from no injurious traction on the fundus through the cord or pressure upon it from above. The author explains the accident as essentially due to a peculiar condition of the placenta, which was about three-fourths of an inch in thickness and lined the whole of the interior of the uterus. This thick placenta must have filled the os in such a manner as to increase its diameter about 1½ inches more than was necessary to permit the escape of the head. This dilatation, conjoined with weakness and flaccidity of the uterine tissues, together with the vis-a-tergo effected by the action of the abdominal muscles, appeared to the author to be the efficient causes of the inversion. Records of several interesting cases of spontaneous inversion occurring prior to period of viability of the fœtus are also included in the paper.

